# Smooth Muscle Myosin Inhibition: A Novel Therapeutic Approach for Pulmonary Hypertension

**DOI:** 10.1371/journal.pone.0036302

**Published:** 2012-05-01

**Authors:** David Ho, Li Chen, Xin Zhao, Nicquanna Durham, Malar Pannirselvam, Dorothy E. Vatner, David J. Morgans, Fady I. Malik, Stephen F. Vatner, You-Tang Shen

**Affiliations:** 1 CV Dynamics, Inc., North Brunswick, New Jersey, United States of America; 2 Cardiovascular Research Institute, University of Medicine and Dentistry of New Jersey, New Jersey Medical School, Newark, New Jersey, United States of America; 3 Cytokinetics, Inc., South San Francisco, California, United States of America; Université de Montréal, Canada

## Abstract

**Objective:**

Pulmonary hypertension remains a major clinical problem despite current therapies. In this study, we examine for the first time a novel pharmacological target, smooth muscle myosin, and determine if the smooth muscle myosin inhibitor, CK-2019165 (CK-165) ameliorates pulmonary hypertension.

**Materials and Methods:**

Six domestic female pigs were surgically instrumented to measure pulmonary blood flow and systemic and pulmonary vascular dynamics. Pulmonary hypertension was induced by hypoxia, or infusion of the thromboxane analog (U-46619, 0.1 µg/kg/min, i.v.). In rats, chronic pulmonary hypertension was induced by monocrotaline.

**Results:**

CK-165 (4 mg/kg, i.v.) reduced pulmonary vascular resistance by 22±3 and 28±6% from baseline in hypoxia and thromboxane pig models, respectively (p<0.01 and 0.01), while mean arterial pressure also fell and heart rate rose slightly. When CK-165 was delivered via inhalation in the hypoxia model, pulmonary vascular resistance fell by 17±6% (p<0.05) while mean arterial pressure and heart rate were unchanged. In the monocrotaline model of chronic pulmonary hypertension, inhaled CK-165 resulted in a similar (18.0±3.8%) reduction in right ventricular systolic pressure as compared with sildenafil (20.3±4.5%).

**Conclusion:**

Inhibition of smooth muscle myosin may be a novel therapeutic target for treatment of pulmonary hypertension.

## Introduction

Pulmonary artery hypertension is a serious and progressive disease of varied etiologies including genetic and environmental factors that also may be associated with other chronic medical conditions [1]. Regardless of the underlying condition, a progressive rise in pulmonary artery pressure and vascular resistance leads to right heart dysfunction and eventual right heart failure, exercise intolerance and ultimately death [2]. Prostacyclin and prostaglandin analogues (e.g., epoprostenol, iloprost, treprostinil), endothelin receptor antagonists (e.g., bosentan, ambrisentan) and phosphodiesterase inhibitors (e.g., sildenafil, tadalafil) are the three major classes of medications currently approved for treatment of pulmonary artery hypertension [1], [3]–[5]. Despite the number of currently available therapeutic options, three year mortality remains high and survival has been estimated at 87%, 76%, and 67% for years 1, 2 and 3 respectively [6]. Accordingly, there remains a continued need for novel therapeutic options to treat pulmonary hypertension. The rationale for this study is to elucidate, for the first time, a novel pharmacological target, pulmonary arterial smooth muscle myosin.

Smooth muscle myosin is responsible for vascular contraction and maintenance of vascular tone [7], [8]; current pulmonary vasodilating mechanisms indirectly inhibit smooth muscle myosin function by acting through second messenger signaling cascades. The relationship between vascular tone and intravascular pressure has been well established. Since the mechanical force generated through the hydrolysis of ATP by smooth muscle myosin is needed for the generation of vascular tone [8], [9], it follows that all regulatory pathways of vascular tone and intravascular pressure ultimately converge at smooth muscle myosin. Recently, selective direct smooth muscle myosin inhibitors capable of blocking ATP hydrolysis and relaxing vascular smooth muscle have been developed and we have reported the efficacy of one such inhibitor, CK-2018448, in a model of systemic hypertension [10]. These smooth muscle myosin inhibitors relax vascular smooth muscle by binding to the myosin enzymatic domain and inhibiting ATP turnover, leaving smooth muscle myosin in a weak actin binding state. However, it is unknown whether smooth muscle myosin inhibitors are able to reduce pulmonary vascular tone in the setting of pulmonary hypertension. In the current study, we examined the therapeutic potential of the smooth muscle myosin inhibitor, CK-2019165 (CK-165, US Patent Application No. 20098/0275537), delivered either as an intravenous infusion or by inhalation of a nebulized solution, in porcine and rodent models of acute pulmonary hypertension. We also compared the effects of pulmonary vascular smooth muscle myosin inhibition with the nitric oxide donor, sodium nitroprusside, the prostacyclin analog, treprostinil, and the phosphodiesterase 5 inhibitor, sildenafil. We also compared the efficacy of CK-165 to sildenafil in a rat model of chronic pulmonary arterial hypertension induced by monocrotaline.

## Results

### Models of Acute Pulmonary Artery Hypertension in Pigs

#### Hypoxia model

**Figure 1:**
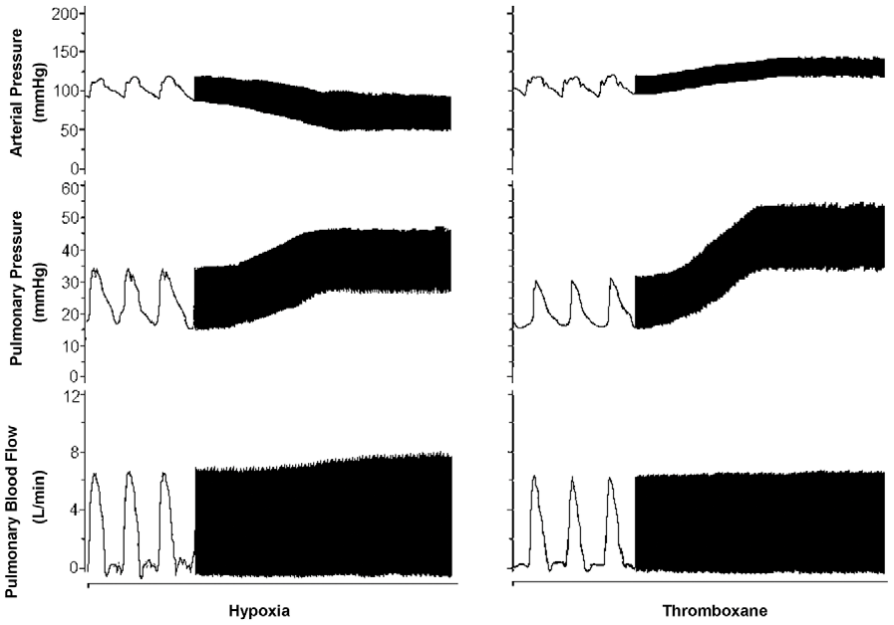
Models of pulmonary hypertension. Hemodynamic recordings of hypoxia and thromboxane pulmonary hypertension models. While the hypoxia resulted in a decrease in arterial pressure and the intravenous infusion of thromboxane resulted in an increase, both methods were able to increase pulmonary pressure with minor effects on pulmonary blood flow.

Hypoxia was achieved by continuous monitoring and titration of the fraction of inspired oxygen to 10%. This resulted in non-significant increases in heart rate and in pulmonary blood flow with a significant 26±1% reduction in mean arterial pressure (p<0.01), a 63±8% increase (p<0.01) in mean pulmonary arterial pressure as well as a 104±20% (p<0.01) increase in pulmonary vascular resistance (Figure 1, Table 1).

**Table 1:** Effects of Hypoxia and Subsequent CK-2019165 or Nitroprusside.

	Pre-Hypertension Baseline	Pulmonary Hypertension baseline	Hypoxia Model (CK-165) % change		Hypoxia Model (Nitroprusside) % change	
	(n = 6)	(n = 6)	Intravenous (n = 5)	Inhalation (n = 6)	Intravenous (n = 6)	Inhalation (n = 6)
**Heart Rate (bpm)**	125±7.7	146±9.4	8.7±1.0%‡	3.3±0.8%‡ ##	8.6±2.7%†	−0.4±0.8%
**Mean Arterial Pressure (mmHg)**	97±3.1	71±1.8**	−10.7±2.8%† ##	−0.6±1.0%	−25.9±2.5%‡	2.5±3.4%
**Mean Pulmonary Artery Pressure (mmHg)**	17.8±0.3	28.8±1.0**	−13.7±2.4%‡	−13.9±3.3%‡	−22.6±3.4%‡	−12.2±1.4%‡
**Pulmonary Blood Flow (L/min)**	2.6±0.1 (n = 5)	2.8±0.2 (n = 5)	9.4±1.1%‡ ##	1.5±1.3% (n = 5)	4.2±2.2% (n = 5)	1.5±1.0% (n = 5)
**Pulmonary vascular resistance (mmHg/L/min)**	4.0±0.3 (n = 5)	8.1±1.0** (n = 5)	−21.9±2.9%‡	−17.2±5.7%† (n = 5)	−14.7±4.7%† (n = 5)	−13.2±3.1%† (n = 5)

* p<0.05 Pulmonary Hypertension vs. Pre-Hypertension Baseline.

** p<0.01 Pulmonary Hypertension vs. Pre-Hypertension Baseline.

† p<0.05 Hypertension Baseline vs. Subsequent CK-2019165 or nitroprusside.

‡ p<0.01 Hypertension Baseline vs. Subsequent CK-2019165 or nitroprusside.

# p<0.05 CK-2019165 vs. nitroprusside.

## p<0.01 CK-2019165 vs. nitroprusside.

#### Thromboxane model

Thromboxane infusion in anesthetized pigs resulted in non-significant changes in mean arterial pressure, heart rate and pulmonary blood flow with a significant 88±8% increase (p<0.01) in mean pulmonary arterial pressure, and a 125±18% increase in pulmonary vascular resistance (p<0.05) (Figure 1, Table 2).

**Table 2:** Effects of Thromboxane and Subsequent CK-2019165 or Nitroprusside in Anesthetized Pigs.

	Pre-Hypertension Baseline	Pulmonary Hypertension baseline	Thromboxane Peak (CK-165) % Change	Thromboxane Peak (Nitroprusside) % Change
	(n = 5)	(n = 5)	(n = 4)	(n = 5)
**Heart Rate (bpm)**	128±4.3	130±6.9	6.7±2.7%	5.1±1.7%†
**Mean Arterial Pressure (mmHg)**	100±5.1	112±6.2	−24.5±2.5%‡ #	−13.6±3.6%‡
**Mean Pulmonary Artery Pressure (mmHg)**	18.5±0.9	34.7±1.4**	−17.4±5.4%†	−15.6±3.0%‡
**Pulmonary Blood Flow (L/min)**	3.2±0.4 (n = 4)	3.0±0.4 (n = 4)	14.85±3.4%† ##	1.42±1.8%
**Pulmonary vascular resistance (mmHg/L/min)**	4.5±0.9 (n = 4)	10.0±1.8* (n = 4)	−28.2±5.5%†	−14.3±4.2%†

* p<0.05 Pulmonary Hypertension vs. Pre-Hypertension Baseline.

** p<0.01 Pulmonary Hypertension vs. Pre-Hypertension Baseline.

† p<0.05 Hypertension Baseline vs. Subsequent CK-2019165 or nitroprusside.

‡ p<0.01 Hypertension Baseline vs. Subsequent CK-2019165 or nitroprusside.

# p<0.05 CK-2019165 vs. nitroprusside.

## p<0.01 CK-2019165 vs. nitroprusside.

### Antihypertensive Effects of CK-165 Compared with Nitroprusside

#### Hypoxia Model of Pulmonary Hypertension

**Figure 2:**
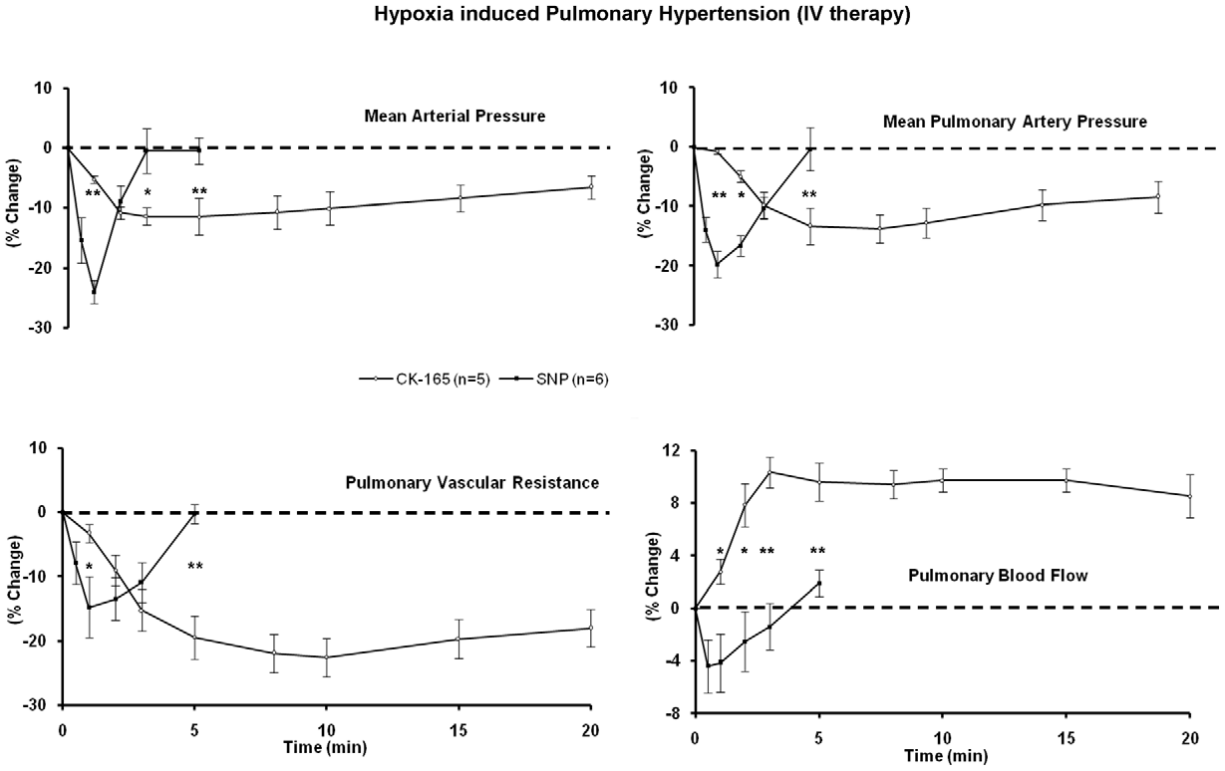
Effects of intravenous CK-165 on hypoxia induced pulmonary hypertension. The efficacy of intravenous CK-165 (4 mg/kg) and nitroprusside (2 µg/kg) were examined and compared in the porcine hypoxia model of pulmonary hypertension. All changes were plotted as percent change from baseline. The relatively longer half life, the more gradual and greater effects on mean pulmonary vascular resistance and blood flow were noted. (* p<0.05, ** p<0.01 CK-165 vs. nitroprusside).

Intravenous delivery of the smooth muscle myosin inhibitor CK-165 (4 mg/kg) in anesthetized pigs, compared with nitroprusside (2 µg/kg) resulted in a lesser peak reduction in mean arterial pressure of 11±3% vs. 26±3% (p<0.01), but with similar peak reductions in mean pulmonary arterial pressure (3.7±1 mmHg vs. 5.8±0.7 mmHg, p = 0.056) and pulmonary vascular resistance (22±3 vs. 15±5%). These results suggest that similar pulmonary vascular effects were obtained with the two drugs, with a lower risk of systemic hypotension with CK-165. Furthermore, a greater increase in peak pulmonary blood flow was seen with CK-165 (9±1%) as compared to nitroprusside (4±2%) (p<0.01) along with a more sustained effect as compared to nitroprusside. (Figure 2, Table 1).

**Figure 3:**
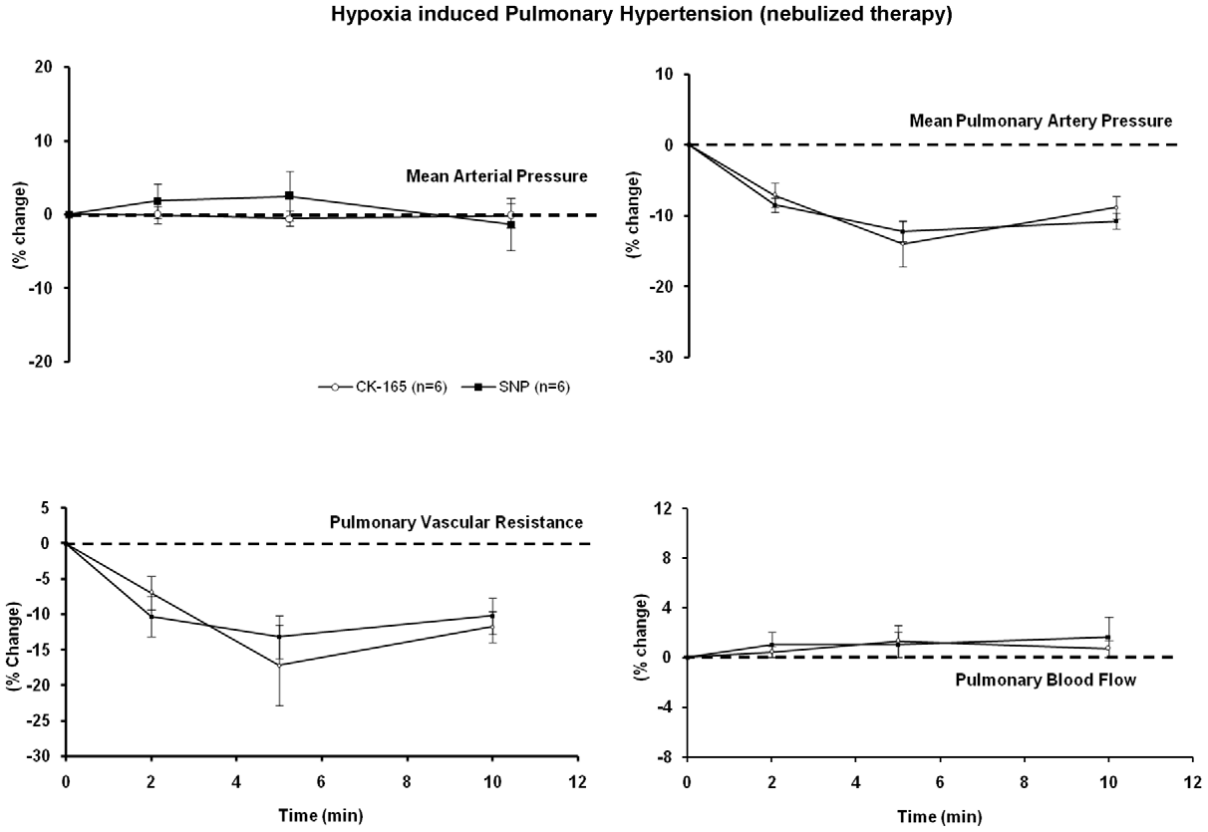
Effects of nebulized CK-165 on hypoxia induced pulmonary hypertension. The efficacy of nebulized CK-165 (4 mg/kg) and nitroprusside (2 µg/kg) were examined and compared in the porcine hypoxia model of pulmonary hypertension. All changes were plotted as percent change from baseline. When delivered continuously, similar effect profiles are observed between CK-165 and nitroprusside.

When CK-165 (10 mg/ml) and nitroprusside (4 mg/ml) were delivered via inhalation in the hypoxia model of pulmonary artery hypertension in anesthetized pigs, they resulted in relatively minor effects on mean arterial pressure, and similar, but modest effects on pulmonary artery pressure and vascular resistance (Figure 3), suggesting that when delivered via inhalation, CK-165 and nitroprusside have similar effects on pulmonary hypertension.

#### Thromboxane Model of Pulmonary Hypertension

**Figure 4:**
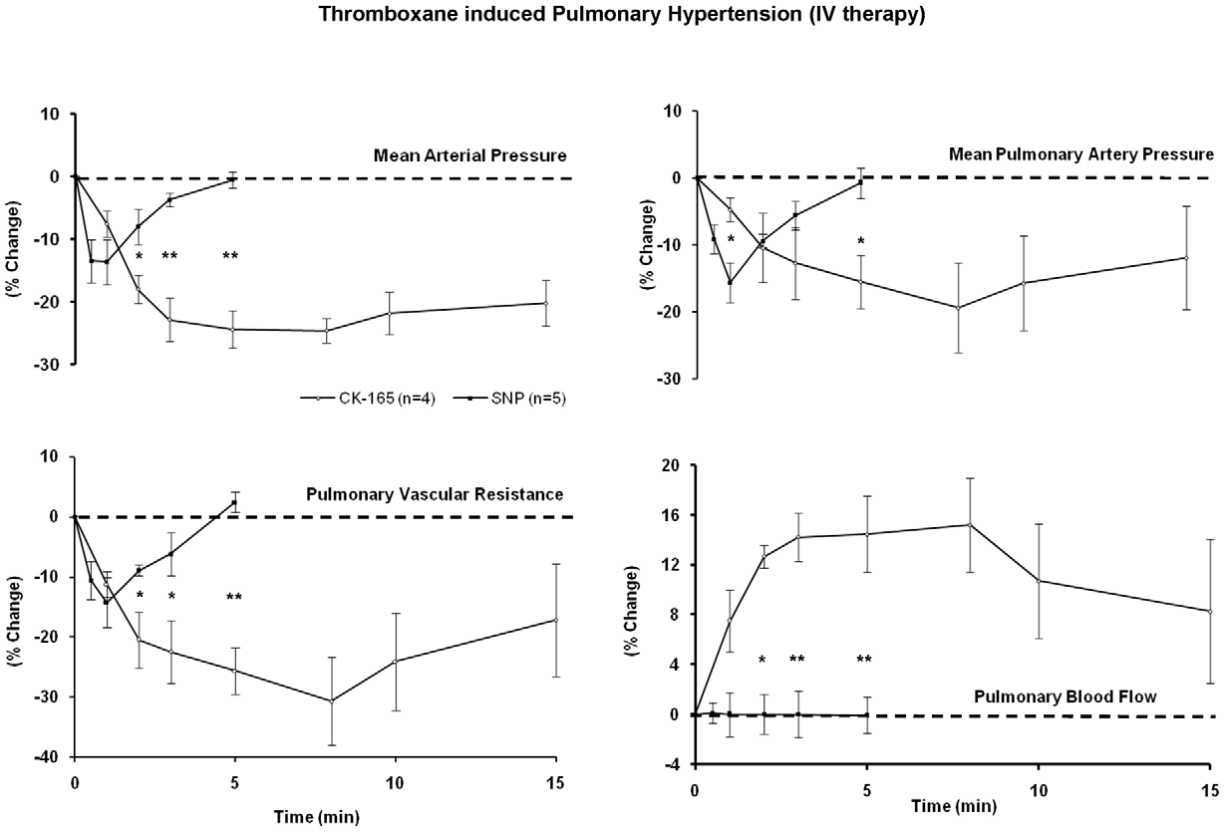
Effects of intravenous CK-165 on thromboxane induced pulmonary hypertension. The efficacy of intravenous CK-165 (4 mg/kg) and nitroprusside (2 µg/kg) were examined and compared in the porcine thromboxane model of pulmonary hypertension. All changes were plotted as percent change from baseline. The relatively longer half life, the more gradual and greater effects on mean arterial pressure, mean pulmonary vascular resistance and blood flow were noted. (* p<0.05, ** p<0.01 CK-165 vs. nitroprusside).

In anesthetized pigs, intravenous delivery of CK-165 resulted in a significantly greater maximal reduction in mean arterial pressure of 25±3% vs. 14±4% (p<0.05) compared with nitroprusside (2 µg/kg). The peak reductions in mean pulmonary arterial pressure 17±5 vs. 16±3% were similar, while the decrease in pulmonary vascular resistance tended to be greater with CK-165 (28±6 vs. 14±4%, p = 0.08), accordingly, as was observed in the conscious pigs, there was a greater increase in peak pulmonary blood flow with CK-165 (15±3%) as compared to nitroprusside (1±2%) (p<0.05). Again the effects of CK-165 were more gradual in onset and longer lasting as compared with nitroprusside (Figure 4, Table 2).

In three conscious pigs, intravenous delivery of CK-165 compared with intravenous delivery of nitroprusside (2 µg/kg) reduced mean arterial pressure (10±2% vs. 8±2%) and mean pulmonary arterial pressure similarly (13±4 vs. 11±1%) while CK-165 decreased pulmonary vascular resistance somewhat more (17±1%) than nitroprusside (6±5%), resulting in a greater increase in peak pulmonary blood flow seen with CK-165 (11±3%) as compared to nitroprusside (1±3%) (p<0.05). Furthermore, the effects of CK-165 were more gradual in onset and longer lasting as compared to the rapid rise and fall seen with nitroprusside (Data not shown).

### Rat Model of pulmonary artery hypertension against treprostinil and sildenafil

**Figure 5:**
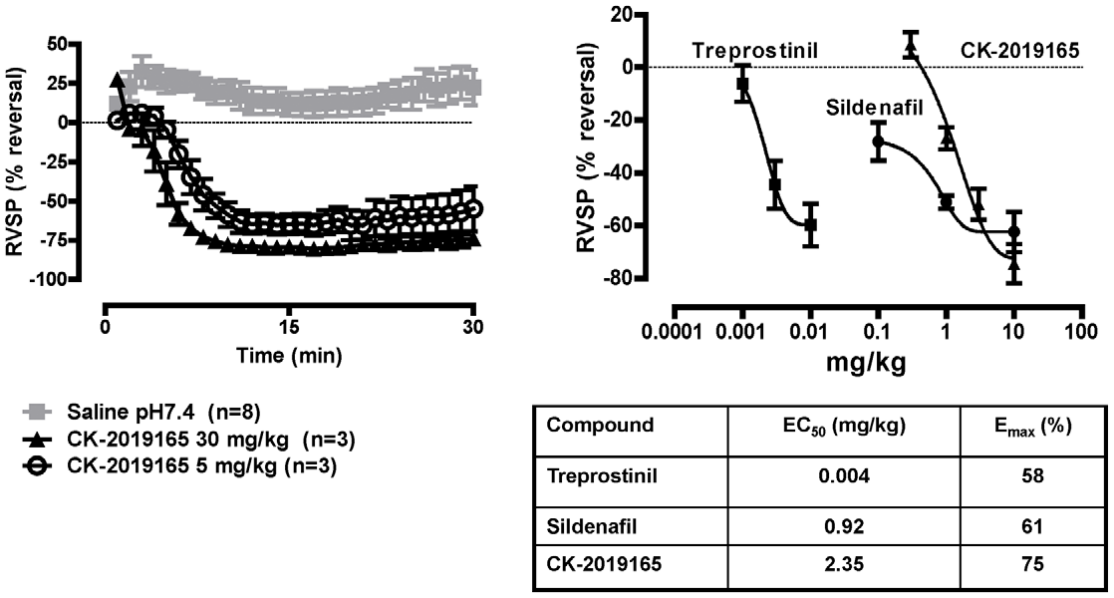
Dose response and pharmacodynamics of CK-165 in rats with U46619 induced pulmonary hypertension. CK-165 decreased RVSP rapidly after nebulization at both 5 mg/kg and 30 mg/kg by 20.4±8.6 and 58.2±6.9% at 6 minutes, respectively (p<0.01 compared with baseline). The maximum decrease in RVSP, E_max_, observed with CK-165 (75±7.53%) is similar to that seen with the prostacyclin analog, treprostinil (58±8.09%) and the phosphodiesterase 5 inhibitor, sildenafil (61±7.65%).

In anesthetized rats, pulmonary hypertension was induced by intravenous infusion of thromboxane (U-46619). Once the right ventricular systolic pressure (RVSP) and mean arterial pressure (MAP) were stable CK-165 was delivered via inhalation at 5 mg/kg (20.4±8.6%, p<0.01) and 30 mg/kg (58.2±6.9%, p<0.01), resulting in a significant reduction in RVSP compared with saline treated rats by the six minute time point (Figure 5). Furthermore, when compared against both the prostacyclin analog treprostinil and the phosphodiesterase 5 inhibitor sildenafil, a similar maximal reduction of RVSP was achieved with CK-165 (Figure 5).

### Model of chronic pulmonary artery hypertension with monocrotaline in rats

**Figure 6:**
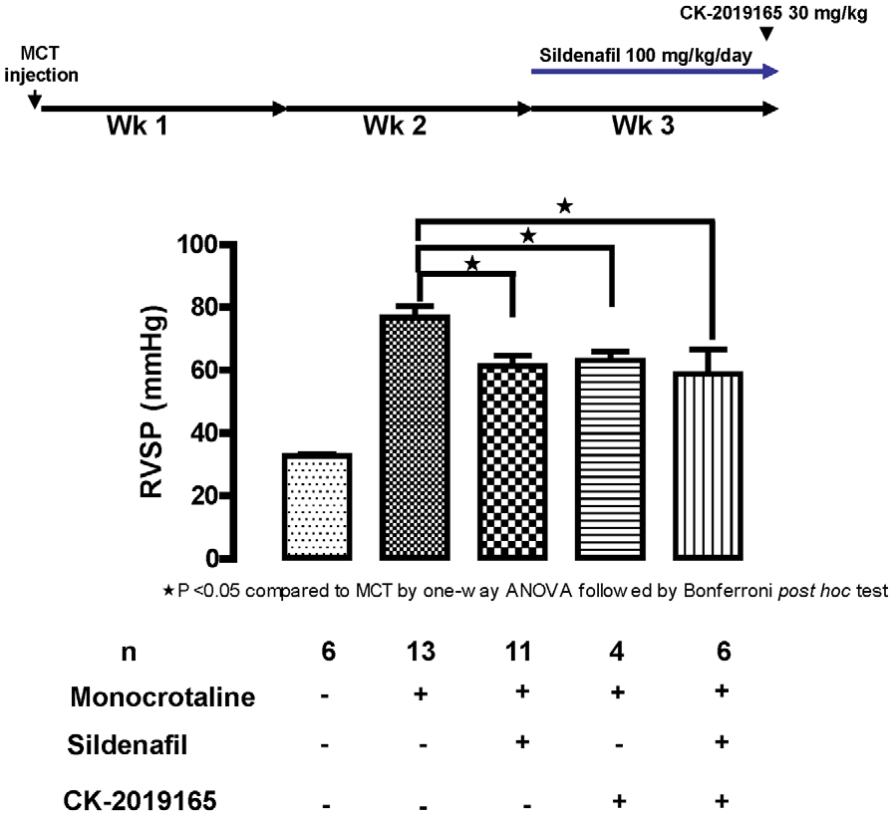
The effect of CK-165 and sildenafil in rats with monocrotaline-induced pulmonary hypertension. CK-165 decreased RSVP following intratracheal nebulization of 30 mg/kg in rats with monocrotaline induced pulmonary artery hypertension by 18.0±3.8% (p<0.05) when compared with vehicle treated group. The decrease is similar to that in monocrotaline rats treated with 100 mg/kg/day sildenafil (20.3±4.5%) for 7 days and treatment with CK-165 does not appear to have additive effects when co-administered with sildenafil.

Pulmonary artery hypertension was induced in rats via administration of a single dose of monocrotaline (60 mg/kg subcutaneously). After two weeks, animals were randomized to receive 100 mg/kg/day sildenafil or no sildenafil for 7 days and at the end of which the groups were randomized again to receive 30 mg/kg CK-165 or placebo. CK-165 was able to induce a similar reduction in RVSP with (20±4.5%, p<0.01) or without (18±3.8%, p<0.05) sildenafil compared with the monocrotaline only group (Figure 6).

### Ex-vivo demonstration of vaso-relaxation via smooth muscle myosin inhibition

**Figure 7:**
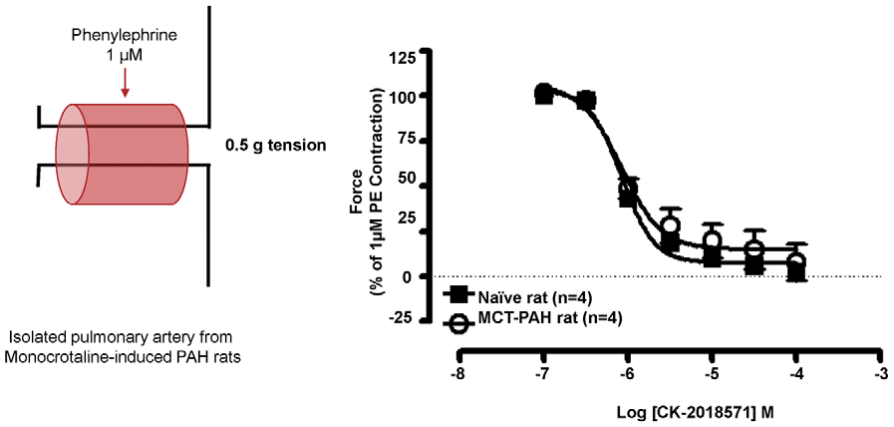
Determination of EC_50_ for CK-2018571 in pulmonary artery tissue rings. CK-2018571 inhibits phenylephrine-induced pulmonary artery contraction in isolated tissue rings from naïve and monocrotaline-treated rats. Pulmonary artery rings were pre-contracted with 1 µM phenylephrine and isometric tension was recorded in the presence of increasing concentrations of CK-2018571. CK-2018571 elicited relaxation in tissues from naïve rats with pEC_50_ = 6.08±0.03, and in tissues from monocrotaline-treated rats with pEC_50_ = 6.08±0.10.

Pulmonary arteries were isolated from naïve rats as well as monocrotaline induced pulmonary artery hypertensive rats and contraction was stimulated via the addition of 1 µM phenylephrine. The addition of CK-2018571 (the parent of the more soluble pro-drug, CK-165) was able to relax phenylephrine induced contraction of pulmonary arterial rings from both naïve and monocrotaline induced pulmonary artery hypertensive rats with a similar EC_50_ of 6.08±0.03 µM and 6.08±0.10 µM, respectively (Figure 7).

## Discussion

Since the final common effector of vascular constriction in pulmonary hypertension is smooth muscle myosin; it follows that an agent that relaxed smooth muscle at the level of smooth muscle myosin would be an effective approach to pulmonary vasodilation. Indeed, this mechanism appears effective in reducing arterial pressure in models of systemic hypertension [10]. The major finding of the current investigation is that the smooth muscle myosin inhibitors, CK-2019165, a soluble pro-drug of CK-2018571 were effective in reducing pulmonary vascular resistance in several models of pulmonary hypertension.

The effectiveness of smooth muscle myosin inhibition was demonstrated in two acute models of pulmonary hypertension (hypoxia and thromboxane) in chronically instrumented pigs with direct and continuous measurements of pulmonary and aortic arterial pressure, pulmonary blood flow and vascular resistance and compared to nitroprusside, a nitric oxide donor examined extensively in animal models of pulmonary hypertension [11]–[14]. Inhalation of nitric oxide is used in patients with pulmonary hypertension to establish the degree of acute reversibility and to reduce pulmonary artery pressure in the setting of adult respiratory distress syndrome without affecting systemic vascular tone [15]. The smooth muscle myosin inhibitor, CK-165, achieved similar efficacy to nitroprusside in these acute models of pulmonary hypertension.

Inhibition of smooth muscle myosin with CK-165 effectively reduced pulmonary artery pressure and pulmonary vascular resistance in both the hypoxia and thromboxane models of pulmonary hypertension regardless of whether the drug was delivered via the intravenous or inhalation route. When the drug was delivered intravenously, there was a fall in systemic arterial pressure suggesting the drug also affected systemic resistance vessels. However, when the drug was delivered through inhalation, this was not observed and the vasodilator effects were localized to the pulmonary vasculature.

A second goal of this study was to compare the effects of CK-165 in acute and chronic rat models of pulmonary hypertension. Smooth muscle myosin inhibition is capable of directly relaxing pulmonary artery vascular rings derived from rats with chronic pulmonary hypertension induced by monocrotaline to a similar degree and potency as those derived from naïve rats, consistent with a direct effect on the pulmonary vasculature. When delivered via nebulization in the thromboxane induced rat model of acute pulmonary hypertension, CK-165 reduced right ventricular systolic pressure (RVSP) consistent with a reduction in pulmonary artery pressure; further, CK-165 was able to reduce the RVSP to a similar extent (although not similar potency) as that seen with either treprostinil or sildenafil.

Finally, to address the question of efficacy in a setting where pulmonary vascular remodeling is present as is found in chronic pulmonary artery hypertension, we examined the efficacy of CK-165 in rats with monocrotaline induced chronic pulmonary artery hypertension. CK-165 reduced RVSP as an acute monotherapy to a similar extent as did 7 days of dosing with sildenafil; the level of reduction in RVSP was essentially the same when co-administrated with sildenafil, suggesting that a maximal effect on RSVP had been attained by each of these therapies.

In conclusion, inhibition of smooth muscle myosin is an effective means to reduce pulmonary vascular resistance and may be a potential therapeutic approach for pulmonary hypertension. Its effects compared with nitroprusside were more gradual and sustained when delivered intravenously, but quite similar when delivered by inhalation. Furthermore, it was able to produce similar effects when compared against both a prostacyclin analog as well as a phosphodiesterase 5 inhibitor in the case of acute pulmonary artery hypertension and against a phosphodiesterase 5 inhibitor in the setting of chronic pulmonary artery hypertension. Based on our results, inhibition of smooth muscle myosin appears to be efficacious in models of pulmonary hypertension. Although the duration of action of the current smooth muscle myosin inhibitor examined in this study may not be sufficient for chronic therapy in this disease, the current study provides proof of concept for this novel therapeutic target and its application to the treatment of pulmonary hypertension.

## Materials and Methods

CK-2019165 and CK-2018571 were provided by Cytokinetics, Inc.

### Animal Models

Animals used in this study were maintained in accordance with the *Guide for the Care and Use of Laboratory Animals of the National Health Institute (NIH, 2010)* and *New Jersey Medical School Institutional Animal Care and Use Committee or by the Institutional Animal Care and Use Committee at Cytokinetics, Inc*. This study has been approved by the *New Jersey Medical School Institutional Animal Care and Use Committee* protocol# 08088 and by the *Cytokinetics, Inc. Institutional Animal Care and Use Committee* under Protocol CK020-09.

#### Pig Models

Six domestic female pigs (15–20 kg) were anesthetized with thiopental (15 mg/kg i.v.) followed by endotracheal intubation and halothane (1.0–1.5 vol%) anesthesia. A left thoracotomy at the 5^th^ intercostal space was performed, Tygon catheters were placed in the descending aorta, pulmonary artery and left atrium to measure their respective pressures. A Tygon catheter was also placed in the right atrium and used for drug administration. A transonic flow probe was placed around the main pulmonary artery to measure pulmonary blood flow. The chest was closed and post-operative analgesics were administered until the animals recovered fully from surgery. Female pigs were chosen over male pigs due to anatomical concerns for animal discomfort while in the sling during the extended experimental time period. All experiments in pigs were performed under anesthesia except for the experiments involving thromboxane induced pulmonary hypertension where experiments were performed in both conscious and anesthetized pigs.

Systemic hemodynamics in pigs were recorded using a Triton system (Triton, Inc.) and PowerLab data acquisition system (ADInstruments, Inc.). Aortic, pulmonary artery and atrial pressure were measured using strain gauge manometers that had been calibrated with a mercury manometer connected to the fluid-filled catheters. Pulmonary blood flow was measured with a Transonic flow meter (Transonic Systems Inc., T206). Pulmonary vascular resistance was calculated: (pulmonary arterial pressure - left atrial pressure)/pulmonary blood flow. Due to instrumentation failure not all hemodynamic variables were recorded for all experiments and animals.

Five to ten days after surgical instrumentation, pulmonary hypertension was induced with two different methods in the same animal at different times. 1) Hypoxic pig model: pigs were intubated, anesthetized with isoflurane, and ventilated at a reduced fraction of inspired O_2_. Pulmonary hypertension resulted after the fraction of inspired O_2_ was reduced to about 10% by adjusting the ratio of N_2_/O_2_ mixture in the inspiratory limb of the ventilator. Pulmonary hypertension was defined as a sustained elevation of mean pulmonary arterial pressure to ≥25 mmHg from an averaged baseline level of 18 mmHg. Arterial oxygen saturation was monitored continuously using a pulse oximeter during the entire experimental period. 2) Thromboxane model: pigs were placed in a sling and infused intravenously with the endoperoxide analog of thromboxane, U46619, at 0.1–0.5 µg/kg/min in the anesthetized state. These pigs were also studied with this procedure in the conscious state. Pulmonary hypertension was defined as a stable pulmonary artery pressure of greater than 30 mmHg. The animals were allowed to breathe spontaneously.

In the hypoxia pig model, once the pulmonary arterial pressure stabilized for 15 min, a volume of 2–3 ml of 10 mg/ml (CK-165) was nebulized over 4–5 min through the endotracheal tube using a Pari LC Plus compressed air nebulizer. The nebulizer was operated at 20 psi which results in an output of ∼4.5 L/min. Sodium nitroprusside (4 mg/ml) was nebulized for the same duration as a control. After the drugs washed out, i.e., pulmonary artery pressure and mean arterial pressure returned to baseline levels and remained stable for 30 min, the smooth muscle myosin inhibitor CK-2019165 (4 mg/kg) was administered intravenously as was the positive control sodium nitroprusside (2 mg/kg). Without prior pharmacokinetic and pharmacodynamic data in the porcine model, the washout period for the animals was determined based on results of hemodynamic monitoring and was determined to be adequate after all hemodynamic variables returned to baseline and were stable for 30 min. In the thromboxane model, CK-165 and nitroprusside were administered intravenously in separate experiments under both the conscious and anesthetized states. In all models systemic and pulmonary vascular changes were monitored and compared.

#### Rat Models

Male Sprague-Dawley rats (Harlan, Indianapolis, IN) weighing between 180 and 210 g were housed in conventional conditions. Acute pulmonary artery hypertension was induced by continuous intravenous infusion of U-46619 (1 µg/kg/min). Chronic pulmonary artery hypertension was induced on day 0 by a single dose of monocrotaline (MCT) (60 mg/kg subcutaneously) (Sigma-Aldrich, St. Louis, MO). The rats were randomly assigned to five experimental groups: 1) Control; 2) MCT; 3) MCT+100 mg/kg/day Sildenafil for 7 days (days 15–21); 4) MCT+CK-2019165 30 mg/kg (on day 21), as a single nebulized dose; and 5) MCT+100 mg/kg Sildenafil (days 15–21)+CK-2019165 30 mg/kg (on day 21), as a single nebulized dose.

Hemodynamic measurements were made following anesthesia with a ketamine, xylazine and acepromazine cocktail at the dose of 80–100 mg/kg, 10 mg/kg and 1 mg/kg respectively, intra-muscular. Rats were mechanically ventilated at 60–80 breaths/minute with a tidal volume of 2.0–3.0 ml/cycle using a Harvard rodent ventilator. The right ventricular pressure was recorded by advancing a 4.0 French pressure sensing catheter (Millar, Houston TX) into the right ventricle via right jugular vein. CK-2019165 was administrated via intra-tracheal nebulization at 30 mg/kg.

For preparation of pulmonary artery tissue rings for *in vitro* study, the main branch of pulmonary artery was isolated from naïve or monocrotaline (MCT)-treated rats and placed in oxygenated physiological salt solution. The pulmonary arteries were cleaned of adherent tissue and cut into rings (2 mm in length) and suspended in isolated tissue baths (volume 10 ml) containing oxygenated Krebs–Ringer bicarbonate buffer with the following composition (in mmol/l): NaCl (118), KCl (4.7), CaCl_2_ (2.5), KH_2_PO_4_ (1.2), MgSO_4_ (1.2), NaHCO_3_ (25), dextrose (11.1) and disodium calcium edetate (0.026), maintained at 37°C and bubbled with 95% O_2_ and 5% CO_2_. A resting tension of 0.5 g was applied (determined by preliminary experiments to afford the optimum tension-length relationship). Following equilibration for 60 minutes, isometric dose response curves were recorded with increasing concentrations of CK-2018571 in rings pre-contracted with phenylephrine (1 µM). Results are mean ± SE, percent of maximum relaxation to CK-2018571 (E_max_) and agonist sensitivity (pEC_50_ = −logEC_50_; EC_50_ is concentration that evokes 50% relaxation). Values were determined using non-linear regression for both naïve and MCT-treated rat pulmonary arterial rings.

### Statistical analysis

Data are expressed as mean±SE. Statistical significance was determined using a paired two tailed t-test when comparing CK-165 to baseline. An unpaired t-test was used to compare the peak responses of CK-165 to nitroprusside. In all experiments, the peak response time was chosen based on continuous monitoring and accordingly, in the CK-165 treated hypoxia model the peak response time was at 5 minutes. Due to a variation in peak times in the CK-165-treated thromboxane model, the average of 5 and 8 minute responses were used as peak for comparative analysis. For nitroprusside in the hypoxia model with inhalation delivery, the peak response time was 5 minutes, in all other pig models the peak response time was 1 minute. For chronic pulmonary artery hypertension studies, the statistical significance was determined by one-way ANOVA followed by Bonferonni's post-hoc test.
